# Effects of letrozole co-treatment on the cumulative live-birth rate among normal responders in gonadotropin-releasing hormone antagonist cycles

**DOI:** 10.3389/fmed.2022.1070583

**Published:** 2022-12-08

**Authors:** Shuyi Zhang, Fumei Gao, Min Fu, Huan Shen, Yanbin Wang, Hongjing Han

**Affiliations:** Reproductive Medical Center, Department of Obstetrics and Gynecology, Peking University People's Hospital, Beijing, China

**Keywords:** cumulative live-birth rate, letrozole, GnRH antagonist, normal responder, ovary stimulation

## Abstract

Studies have shown that letrozole cotreatment can improve clinical outcomes in high and poor responders in GnRH-antagonist protocol. However, whether letrozole is also beneficial to normal responders is not known. To investigate the clinical value of letrozole cotreatment during ovarian stimulation *in vitro* fertilization for normal ovarian reserve patients who were treated with the GnRH antagonist protocol, we conducted a retrospective study that based data from 1 January to 31 December 2017 for all IVF–ICSI GnRH-antagonist protocols. A total of 252 women who aged <40 years, FSH <10 IU/L on day 3 and antral follicle counting (AFC) >6 were included in the analysis (96 in the letrozole group and 156 in the no-letrozole group). The cumulative live-birth rate was calculated as the first live birth achieved after all cycles having an embryo transfer (cycles using fresh embryos and frozen–thawed embryos) among both groups. The initial gonadotropin (Gn) dosage and total Gn dosage were significantly lower and the number of days of Gn treatment was significantly fewer in the letrozole group than the non-letrozole group (*p* < 0.05). There were also significant between-group differences in luteinizing hormone, estradiol, and progesterone concentrations; and the number of metaphase II oocytes on the day of human chorionic gonadotropin treatment (*p* < 0.05). There was a significant difference in the implantation rate between the two groups that the letrozole group higher than the non-letrozole group (39.79 vs. 27.96%, *p* = 0.006), but there was no significant difference in the cumulative live-birth rate. The combination of letrozole with a GnRH antagonist may have no effect on the clinical pregnancy rate or cumulative live-birth rate in patients with a normal ovarian reserve. However, letrozole may increase the rate of embryo implantation and may reduce the requirement for exogenous gonadotrophins and, consequently, the cost of an IVF treatment cycle. In addition, the decreased estradiol level in the ovarian simulation by letrozole supports letrozole can be a safe solution for fertility preservation in estrogen-related cancer patients.

## Introduction

Letrozole, a third-generation aromatase inhibitor, binds the heme group of cytochrome p450, blocks the conversion of androstenedione to estrone and testosterone to estradiol (E2), and significantly reduces circulating estrogen concentrations, without affecting estrogen receptors in peripheral tissues (1). Letrozole induces a >86% decrease in the plasma estrone concentration and a ≥67% reduction in the circulating E2 concentration after 14 days of administration. It was first administered orally to postmenopausal patients with advanced breast cancer in 1996 to reduce the amount of estrogen produced by peripheral androgen aromatization (2, 3). The characteristics of letrozole, with its oral route of administration, low cost, relatively short half-life, and absence of antiestrogenic effects, make it a promising drug for ovulation induction. In 2,000, letrozole was first used to induce ovulation in anovulatory women with polycystic ovary syndrome (PCOS). It successfully induced ovarian follicle development but did not affect endometrial thickness (4). Thereafter, letrozole was widely used for intrauterine insemination and in various protocols of ovarian stimulation for *in vitro* fertilization/intracytoplasmic sperm injection (IVF/ICSI) for hyper-responders and poor responders. In a retrospective analysis of hyper-responders receiving an IVF GnRH antagonist protocol, the average E2 concentration and the E2 concentration of a single follicle on the day of human chorionic gonadotropin (hCG) treatment were significantly lower in the letrozole group (*n* = 93) than the non-letrozole group (*n* = 86). However, the incidence of ovarian hyperstimulation syndrome was significantly lower in the letrozole group (3.20 vs. 5.80%, *p* = 0.000) than the non-letrozole group (5, 6). Thus, letrozole is used as the first-line ovulation stimulation drug for patients with PCOS (7). For poor responders, a retrospective cohort study showed that the number of oocytes retrieved was significantly higher in the letrozole group than the non-letrozole group (5.30 ± 2.0 vs. 4.30 ± 1.90, *p* = 0.020), while the total dose was significantly lower and the number of days of recombinant human follicle stimulating hormone (FSH) administration were significantly fewer in the letrozole group (4). Furthermore, a recent meta-analysis of 31 studies showed that the live-birth rate in poor responders significantly increased by 7% after letrozole co-treatment (8). However, the effects of letrozole co-treatment on pregnancy outcomes for normal responders undergoing IVF are not clear. In addition, previous studies have not focused on the cumulative live-birth rate, which provides an all-inclusive success rate for assisted reproductive technology (ART) (9).

Therefore, the present study was performed to investigate whether letrozole co-treatment could improve the cumulative live-birth rate for normal responders to GnRH antagonist treatment during IVF/ICSI cycles.

## Materials and methods

### Ethical approval of the study protocol

This study was approved by the Reproductive Ethics Committee of the Peking University Peoples' Hospital (Beijing, China). All treatments were administered in accordance with the Declaration of Helsinki, 1964 and its later amendments.

### Study design

In this retrospective study, medical records from January 1 to December 31, 2017 were reviewed for all IVF/ICSI cycles involving a GnRH antagonist protocol at the Reproductive Center of Peking University Peoples' Hospital.

### Inclusion criteria

Women were included in the study if they: (i) were aged <40 years; (ii) had an FSH concentration <10 IU/L on day 3 of the menstruation; and (iii) had an antral follicle count (AFC) >6. To evaluate the effect of letrozole on the cumulative live-birth rate, only women who achieved their first live birth or had used all of the fresh and frozen embryos acquired from a GnRH-antagonist-stimulated cycle were included.

### Exclusion criteria

Women were excluded from the study if they: (i) had occult ovarian failure (FSH concentration ≥10 IU/L on day 3 of the menstruation); (ii) had received clomiphene to stimulate ovulation; or (iii) had an endocrine disorder (PCOS, diabetes mellitus, thyroid dysfunction, congenital adrenal hyperplasia, hyperprolactinemia, or Cushing syndrome) or a uterine anomaly confirmed by hysterosalpingography or hysteroscopy; (iiii) have suffering from autoimmune diseases diagnosed by specialist physician (systemic lupus erythematosus, dermatomyositis, connective tissue disease etc.). One thousand and twenty-one patients who received a GnRH antagonist protocol for IVF/ICSI and had cumulative pregnancy outcomes and 252 patients with a normal response to GnRH antagonist treatment were included in the analysis.

### Protocols for ovarian stimulation

All participants received a flexible protocol involving a GnRH antagonist for controlled ovarian hyperstimulation (COH). None of the participants received an oral contraceptive pill before commencing the IVF cycle. Ovarian stimulation with recombinant FSH (150–225 IU daily; Gonal-F; Merck Serono, Coinsins, Switzerland) began on day 2 of the menstrual cycle and continued for 3 consecutive days. The starting dose was determined using the patients' age, ovarian reserve, body mass index (BMI), and previous response to COH. On the initiation day (D2), 5 mg of letrozole (Furui; Jiangsu Hengrui Medicine, Lianyungang, China) was administered daily for 5 consecutive days. The recombinant FSH dose was then adjusted according to the serum E2 concentration and follicular growth, as monitored by serial transvaginal ultrasound. Administration of the GnRH antagonist (0.25 mg of ganirelix or cetrorelix administered at 10 a.m. daily) was initiated based on a flexible protocol (in general, when the lead follicle was 13–14 mm in diameter) and continued until the day of hCG administration. When ≥2 leading follicles had reached 18 mm in diameter, final oocyte maturation was triggered by the administration of 250 mg of recombinant hCG (Ovidrel; Merck Serono, Geneva, Switzerland) alone, which was equivalent to ~6,500 IU of hCG according to the manufacturer's data, or 0.2 mg of triptorelin (Ferring International Center, SaintPrex, Switzerland) plus 2,000 IU of hCG (Livzon, Zhuhai, China). Oocyte retrieval was performed by transvaginal ultrasonography 35–37 h later. ICSI was performed for patients experiencing severe male-factor infertility.

### Embryo culture

Fertilization (i.e., the appearance of two distinct pronuclei and two polar bodies) was assessed 16–18 h after insemination. The embryo culture and embryo evaluation criteria were referred to the published research in our center (10).

### Embryo transfer

Fresh embryos were transferred 3 days after oocyte retrieval. In frozen–thawed embryo transfer (FET) cycles, the embryos were transferred in natural cycles or in hormonal replacement cycles. Women who failed to ovulate were given estradiol valerate (Progynova^®^; 3 mg, p.o., b.d.; Bayer, Leverkusen, Germany) from days 2 to 3 of the menstrual cycle. Progesterone (60 mg, once-daily intramuscular injection) was administered when the thickness of the endometrium was ≥8 mm. One or two embryos were transferred depending on embryo quality and the patient's age.

### Support during the luteal phase

For fresh embryo transfer cycles, support during the luteal phase comprised daily intramuscular injection of 40 mg of progesterone in oil (Xianju, Taizhou, China) along with oral supplementation with 30 mg of dydrogesterone (Duphaston; Abbot Biologicals, Olst, the Netherlands), starting on the day of oocyte retrieval. For FET cycles, intramuscular injection of 60 mg of progesterone in oil (Xianju) was performed from the day of endometrial transformation. The serum β-hCG concentration was measured 14 days after embryo transfer, and a value >5 IU/mL was considered to denote clinical pregnancy. Luteal support continued until 10 weeks of pregnancy.

### Outcome variables

The primary outcome measure was the cumulative live-birth rate, which was calculated as the number of women who achieved their first live birth (>28 weeks of gestation) in fresh embryo transfer cycles or in subsequent FET cycles divided by the number of patients in the two groups. The number of oocytes retrieved; the number of metaphase II (MII) oocytes; and the rates of fertilization, implantation, clinical pregnancy, and miscarriage were also analyzed. The definitions of these metrics are described in our previous article (10).

### Statistical analyses

Data analyses were performed using SPSS 24.0 (IBM, Armonk, NY, USA). The Shapiro–Wilk test was used to determine the normality of the data distribution. Based on the results of this determination, parametric tests were performed. Continuous variables are presented as the mean ± standard deviation and were compared using Student's *t*-test or the Mann–Whitney *U*-test. Categorical variables are presented as frequencies and percentages. A chi-square test was used for comparisons between groups. *p* < 0.05 was considered significant.

## Results

Two hundred and fifty-two women met the inclusion criteria and were included in the analysis (96 in the letrozole group and 156 in the non-letrozole group). The baseline age; body mass; BMI; number of years of infertility; basic FSH, luteinizing hormone (LH), and E2 concentrations; AFC; and demographic characteristics did not differ significantly between the groups (Table 1). The initial Gn dosage and total Gn dosage were significantly lower and the number of days of Gn treatment were significantly fewer in the letrozole group than the non-letrozole group (*p* < 0.05), as shown in Table 1. The E2 concentration on the day of hCG administration was significantly lower in the letrozole group than the non-letrozole group (1,523.84 ± 1,050.53 vs. 2,743.89 ± 1,332.06, *p* < 0.0001), while the LH and progesterone concentrations were higher in the letrozole group (Table 1). However, the endometrial thickness on the day of hCG administration was similar between the two groups. In terms of the outcomes of ovulation induction, the number of MII oocytes was higher in the letrozole group than the non-letrozole group (8.97 ± 4.22 vs. 7.74 ± 3.94, *p* = 0.022), but the numbers of two-pronuclear embryos, blastocysts, and high-quality embryos were similar between the two groups (Table 2).

**Table 1:** Baseline patient characteristics for the two groups.

	**Letrozole (*n* = 96)**	**Non-letrozole (*n* = 156)**	***P***
Age (years)	32.89 ± 3.76	32.56 ± 3.71	0.507
Body mass (kg)	59.37 ± 8.39	58.54 ± 8.18	0.435
BMI (kg/m^2^)	22.59 ± 2.96	22.31 ± 2.88	0.466
Duration of infertility (years)	3.40 ± 2.46	3.77 ± 3.06	0.320
FSH (IU/L) at day 3	7.50 ± 1.66	7.22 ± 1.58	0.174
LH (IU/L) at day 3	4.20 ± 2.44	4.14 ± 3.18	0.867
E2 (IU/L) at day 3	41.57 ± 25.31	62.74 ± 171.24	0.230
AFC	11.31 ± 4.41	11.58 ± 4.07	0.628
**Cause of infertility**			
Male factor	26 (27.08%)	37 (23.72%)	0.110
Tubal factor	37 (38.54%)	55 (35.26%)	0.123
Ovulation dysfunction	3 (3.13%)	6 (3.85%)	0.205
Endometriosis	7 (7.29%)	9 (5.77%)	0.079
Combined	18 (18.75%)	42 (26.92%)	0.242
Unexplained	5 (5.21%)	7 (4.49%)	0.105
**Fertilization**			
IVF	54 (56.25%)	91 (58.33%)	0.159
ICSI	42 (43.75%)	65 (41.67%)	0.135

Values are the mean ± standard deviation or percentage. BMI, body mass index; FSH, follicle-stimulating hormone; LH, luteinizing hormone; E2, estradiol; AFC, antral follicle count; IVF, *in vitro* fertilization; ICSI, intracytoplasmic sperm injection.

**Table 2:** Comparison between letrozole and non-letrozole groups: characteristics of ovarian stimulation.

	**Letrozole (*n* = 96)**	**Non-letrozole (*n* = 156)**	***P***
Initial dose of gonadotropin (IU)	180.08 ± 57.99	221.63 ± 52.13	< 0.0001
Total dose of gonadotropins (IU)	1,951.95 ± 639.42	2,197.60 ± 636.87	0.003
Duration of stimulation (d)	9.19 ± 1.50	9.69 ± 1.59	0.013
Endometrial thickness (mm) on trigger day	9.56 ± 2.27	9.59 ± 2.43	0.919
E2 concentration (ng/mL) on trigger day	1,523.84 ± 1,050.53	2,743.89 ± 1,332.06	< 0.0001
LH concentration (ng/mL) on trigger day	4.78 ± 4.33	2.38 ± 1.79	< 0.0001
Progesterone concentration (ng/mL) on trigger day	1.45 ± 0.73	1.17 ± 0.69	0.003
No. of oocytes retrieved	11.31 ± 4.19	11.23 ± 4.32	0.883
No. of MII oocytes	8.97 ± 4.22	7.74 ± 3.94	0.022
Fertilization rate (%)	81.54 ± 18.12	84.56 ± 16.46	0.175
No. of 2PN embryos	7.02 ± 3.42	7.49 ± 3.45	0.297
No. of embryos available	2.44 ± 1.08	2.28 ± 1.09	0.273
No. of high-quality embryos	1.23 ± 1.16	1.19 ± 1.16	0.775
No. of blastocysts	1.99 ± 1.96	2.04 ± 1.91	0.845

E2, estrogen; LH, luteinizing hormone; MII, metaphase II; 2PN, two-pronuclear.

Thirty-three women underwent fresh embryo transfer in the letrozole group compared with 64 women in the non-letrozole group. A higher abortion rate after fresh embryo transfer cycles was observed in the letrozole group, although this difference was not significant (20.00 vs. 4.65%, *p* = 0.058; Table 3). The implantation, clinical pregnancy, and live-birth rates after fresh embryo transfer cycles were similar between the two groups (Table 3). Further, the cumulative pregnancy outcomes of the two groups were assessed. The implantation rate was significantly higher in the letrozole group than the non-letrozole group (39.79 vs. 27.96%, *p* = 0.006). However, there was no significant difference in the abortion, cumulative pregnancy, or cumulative live-birth rates between the two groups (Table 4).

**Table 3:** Reproductive outcomes of fresh-embryo transfer cycles in the study group.

	**Letrozole (*n* = 33)**	**Non-letrozole (*n* = 64)**	***P***
Implantation rate	38.37% (33/86)	32.51% (53/163)	0.355
Clinical-pregnancy rate per ET	75.75% (25/33)	67.18% (43/64)	0.382
Abortion rate	20.00% (5/25)	4.65% (2/43)	0.058
Live-birth rate per ET	60.60% (20/33)	60.06% (41/64)	0.738

ET, embryo transfer.

**Table 4:** Cumulative reproductive outcomes in the study groups.

	**Letrozole** **(*n* = 96)**	**Non-letrozole** **(*n* = 156)**	***P***
Implantation rate	39.76% (103/259)	27.96% (111/397)	0.006
Clinical-pregnancy rate per ET	71.85% (69/96)	70.51% (110/156)	0.817
Abortion rate	17.39% (12/69)	10.91% (12/110)	0.215
Cumulative live-birth rate	72.92% (70/96)	71.15% (111/156)	0.714

ET, embryo transfer.

## Discussion

In this study, we investigated the effect of letrozole co-treatment on the cumulative live-birth rate among normal responders to a GnRH antagonist IVF protocol. The results demonstrated that although letrozole co-treatment did not affect the cumulative live-birth rate of normal responders (72.92 vs. 71.15%, *p* = 0.71), the number of oocytes retrieved was significantly higher (8.97 ± 4.22 vs. 7.74 ± 3.94, *p* = 0.022) in the letrozole group than the non-letrozole group.

The E2 concentration on the day of hCG administration was significantly lower in the letrozole group than the non-letrozole group. In addition, the initial dose of Gn, the total dose of Gn, and the duration of stimulation were also significantly reduced in the letrozole group compared to the non-letrozole group. Patients in the letrozole group were administered 5 mg of letrozole daily for 5 days from the day that ovarian stimulation was initiated. The E2 concentration on the day of hCG administration was 44% lower in the letrozole group (1,523.84 ± 1,050.53 IU) than the non-letrozole group (2,743.89 ± 1,332.06 IU). This result was similar to the results of a double-blinded, placebo-controlled, randomized study of letrozole or placebo interventions during ovarian stimulation for IVF treatment, which showed that daily administration of 5 mg of letrozole significantly suppressed E2 concentrations in the follicular phase by 58% (11). This was very important for the patients with estrogen-related malignancies such as breast cancer and endometrial cancer (12). During these patients ovarian stimulation for oocyte or embryo freezing should be typically avoided, because exposure to the supraphysiological estrogen levels may not be safe. But the results in the present study showed that letrozole could significantly reduce the peak estradiol level in the ovarian stimulation without affecting the cumulative live birth. Thus, it was recommended that letrozole can be a safe solution for fertility preservation in estrogen-related cancer patients (13). Compared to d-chiro-inositol, which modulates the activity of aromatase by reducing the corresponding gene expression to suppressed E2 concentrations (14), letrozol inhibits the enzyme activity by blocking the active site (15). As a synthetic third-generation aromatase inhibitor that specifically blocks the conversion of androgen to estrogen (16), letrozole may cause a temporary accumulation of intraovarian androgens. It has been shown that androgens promote the initiation of primordial follicle growth and increase the number of growing preantral and small antral follicles in the ovaries (17, 18). In addition, androgen accumulation in the follicle may stimulate insulin-like growth factor, which may synergize with FSH to promote folliculogenesis (19), IGF2 addition increased follicle survival rates, diameters and inhibin B production, as well as granulosa cell proliferation (20). Thus, letrozole increases ovarian sensitivity to gonadotropins, which may have contributed to the decrease in the total Gn dose and the duration of stimulation in the letrozole group in the present study. The results of our study were consistent with those of other studies investigating the effect of letrozole during IVF treatment. A retrospective study including 303 women revealed a reduction of 1.10 days of the duration of stimulation in the letrozole group compared to the non-letrozole group (8).

It was also found that the LH and progesterone concentrations on the day of hCG administration were higher in the letrozole group than the non-letrozole group. Increased endogenous production of LH has previously been described as a result of decreased E2 feedback from the hypothalamic–pituitary axis (21, 22). The higher LH concentrations observed in the luteal phase after letrozole co-treatment increase progesterone concentrations in patients treated with letrozole, but this does not continue into the luteal phase (23).

We used the cumulative live-birth rate as the primary outcome for the first time to evaluate the effect of letrozole on GnRH antagonist treatment. The cumulative birth rate has been shown to provide an all-inclusive success rate for ART (10)and was described in detail in our previous study (10). In the present study, all patients were followed up for 3 years. The cumulative live-birth rate was similar in the letrozole (72.92%, 70/96) and non-letrozole (71.15%, 111/156) groups (Table 4). To comprehensively understand the effect of letrozole on pregnancy outcomes, we further explored the outcomes of FET cycles. Thirty-three patients underwent FET cycles in the letrozole group, and 64 patients underwent FET cycles in the non-letrozole group. In FET cycles, the live-birth rate was similar between the two groups. However, a significantly higher abortion rate was observed in the letrozole group than the non-letrozole group (20.0 vs. 4.65%, *p* = 0.058). The higher abortion rate may be due to imbalanced endocrine hormone concentrations in the luteal phase induced by letrozole treatment. It has previously been shown that the E2 concentration decreases and the progesterone concentration increases in the luteal phase after letrozole co-treatment (11). The altered balance between E2 and progesterone in the luteal phase, as seen in the present study, may have a favorable effect on the endometrium and implantation window and may reduce the need for exogenously administered progesterone to sustain endometrial development (11). However, given the cost of letrozole and Gn and the total Gn dosage in the two groups, letrozole co-treatment as part of an IVF GnRH antagonist protocol may effectively reduce the economic burden on patients, which is of great significance in health economics.

The strengths of our study were: (i) a large patient cohort; (ii) a long follow-up period; and (iii) the use of the cumulative live-birth rate as the primary outcome in the two groups, which provided an all-inclusive success rate for ART. The main limitations of our study were its retrospective design, its non-originality, and the possibility of selection bias.

## Conclusion

In conclusion, the combination of letrozole and a GnRH antagonist regimen for ovulation induction did not affect the cumulative live-birth rate in patients with a normal ovarian reserve, but it increased the number of oocytes retrieved, decreased the E2 concentration on the day of hCG administration, and decreased the total Gn consumption. The decreased E2 level in the ovarian simulation supports letrozole can be a safe solution for fertility preservation in estrogen-related cancer patients. The lower requirement for exogenous gonadotrophins consequently reduces the economic burden on patients, which is of great significance in health economics.

## Data availability statement

The original contributions presented in the study are included in the article/supplementary material, further inquiries can be directed to the corresponding author/s.

## Ethics statement

This study was approved by the Reproductive Ethics Committee of the Peking University Peoples' Hospital (Beijing, China). The patients/participants provided their written informed consent to participate in this study.

## Author contributions

YW and HH conceived and designed this study. SZ and FG contributed to the acquisition and analyses of the data. SZ also contributed to the draft of the manuscript. MF and HS were responsible for data collection. FG and HH contributed to revising the manuscript. All authors contributed to the article and approved the submitted version.

## References

[1] Bezerra Espinola MS Lagana AS Bilotta G Gullo G Aragona C Unfer V. D-chiro-inositol induces ovulation in non-polycystic ovary syndrome (PCOS), non-insulin-resistant young women, likely by modulating aromatase expression: a report of 2 cases. Am J Case Rep. (2021) 22:e932722. 10.12659/AJCR.932722 34615846 PMC8503791

[2] Bisagni G Cocconi G Scaglione F Fraschini F Pfister C Trunet PF. Letrozole, a new oral non-steroidal aromastase inhibitor in treating postmenopausal patients with advanced breast cancer. A pilot study oncology. Ann Oncol. (1996) 7:99–102. 10.1093/oxfordjournals.annonc.a010490 9081401

[3] Smith IE Dowsett M. Aromatase inhibitors in breast cancer. N Engl J Med. (2003) 348:2431–42. 10.1056/NEJMra023246 12802030

[4] Mitwally MF Casper RF. Aromatase inhibition for ovarian stimulation: future avenues for infertility management. Obstet Gynecol. (2002) 2002:255–63. 10.1097/00001703-200206000-00003 12032380

[5] Ecemis T Tasci Y Caglar GS. Controlled ovarian hyperstimulation with sequential letrozole co-treatment in normo/high responders. Gynecol Endocrinol. (2016) 32:206–9. 10.3109/09513590.2015.1110133 26487376

[6] Ming HZS Qun L. Application of letrozole in ovarian stimulation for hyperresponders. Chin J Clin Obstet Gynecol. (2021) 22:394–7. 10.13390/j.issn.1672-1861.2021.04.016

[7] Legro RS Brzyski RG Diamond MP Coutifaris C Schlaff WD Casson P. Letrozole versus clomiphene for infertility in the polycystic ovary syndrome. N Engl J Med. (2014) 371:119–29. 10.1056/NEJMoa1313517 25006718 PMC4175743

[8] Bülow NS Holt MD Skouby SO Petersen KB Englund AL Pinborg A. Co-treatment with letrozole during ovarian stimulation for IVF/ICSI: a systematic review and meta-analysis. Reprod Biomed Online. (2022) 44:717–36. 10.1016/j.rbmo.2021.12.006 35183444

[9] Zegers-Hochschild F Adamson GD Dyer S Racowsky C de Mouzon J Sokol R. The international glossary on infertility and fertility care, 2017. Human Reprod. (2017) 32:1786–801. 10.1093/humrep/dex234 29117321 PMC5850297

[10] Gao F Wang Y Fu M Zhang Q Ren Y Shen H. Effect of a “dual trigger” using a GnRH agonist and hCG on the cumulative live-birth rate for normal responders in GnRH-antagonist cycles. Front Med. (2021) 8:683210. 10.3389/fmed.2021.683210 34113641 PMC8185054

[11] Poulsen LC Warzecha AK Bülow NS Bungum L Macklon NS Yding Andersen C. Skouby effects of letrozole cotreatment on endocrinology and follicle development in women undergoing ovarian stimulation in an antagonist protocol. Hum Reprod. (2022) 37:1557–71. 10.1093/humrep/deac119 35652260

[12] Zaami S Melcarne R Patrone R Gullo G Negro F Napoletano G. Oncofertility and reproductive counseling in patients with breast cancer: a retrospective study. J Clin Med. (2022) 11. 10.3390/jcm11051311 35268402 PMC8911138

[13] Carvalho BR Rodrigues JK Campos JR Silva AA Marinho RM Silva A. Strategies to preserve the reproductive future of women after cancer. JBRA Assist Reprod. (2014) 18:16–23. 10.5935/1518-0557.20140087 35761719 PMC9237914

[14] Gambioli R Forte G Aragona C Bevilacqua A Bizzarri M Unfer V. The use of D-chiro-Inositol in clinical practice. Eur Rev Med Pharmacol Sci. (2021) 25:438–46. 10.26355/eurrev_202101_24412 33506934

[15] Quaas AM Legro RS. Pharmacology of medications used for ovarian stimulation. Best Pract Res Clin Endocrinol Metab. (2019) 33:21–33. 10.1016/j.beem.2018.10.002 30470497

[16] Skriver SK Laenkholm AV Rasmussen BB Handler J Grundtmann B Tvedskov TF. Neoadjuvant letrozole for postmenopausal estrogen receptor-positive, HER2-negative breast cancer patients, a study from the danish breast cancer cooperative group (DBCG). Acta Oncol. (2018) 57:31–7. 10.1080/0284186X.2017.1401228 29168427

[17] Vendola K Zhou J Wang J Famuyiwa OA Bievre M Bondy CA. Androgens promote oocyte insulin-like growth factor I expression and initiation of follicle development in the primate ovary. Biol Reprod. (1999) 61:353–7. 10.1095/biolreprod61.2.353 10411511

[18] Weil SJ Vendola K Zhou J Adesanya OO Wang J Okafor J. Androgen receptor gene expression in the primate ovary: cellular localization, regulation, and functional correlations. J Clin Endocrinol Metab. (1998) 83:2479–85. 10.1210/jcem.83.7.4917 9661631

[19] Orisaka M Miyazaki Y Shirafuji A Tamamura C Tsuyoshi H Tsang BK. The role of pituitary gonadotropins and intraovarian regulators in follicle development: a mini-review. Reprod Med Biol. (2021) 20:169–75. 10.1002/rmb2.12371 33850449 PMC8022101

[20] Tkachenko OY Wolf S Lawson MS Ting AY Rodrigues JK Xu F. Insulin-like growth factor 2 is produced by antral follicles and promotes preantral follicle development in macaquesdagger. Biol Reprod. (2021) 104:602–10. 10.1093/biolre/ioaa227 33348377 PMC7962767

[21] Xi W Liu S Mao H Yang Y Xue X Lu X. Use of letrozole and clomiphene citrate combined with gonadotropins in clomiphene-resistant infertile women with polycystic ovary syndrome: a prospective study. Drug Des Devel Ther. (2015) 9:6001–8. 10.2147/DDDT.S83259 26648691 PMC4651359

[22] Yun BH Chon SJ Park JH Seo SK Cho S Choi YS. Minimal stimulation using gonadotropin combined with clomiphene citrate or letrozole for intrauterine insemination. Yonsei Med J. (2015) 56:490–6. 10.3349/ymj.2015.56.2.490 25684000 PMC4329363

[23] Bulow NS Skouby SO Warzecha AK Udengaard H Andersen CY Holt MD. Impact of letrozole co-treatment during ovarian stimulation with gonadotrophins for IVF: a multicentre, randomized, double-blinded placebo-controlled trial. Hum Reprod. (2022) 37:309–21. 10.1093/humrep/deab249 34792133

